# Application of Extracorporeal Membrane Oxygenation in Patients With Severe Acute Respiratory Distress Syndrome Caused by *Pneumocystis jirovecii* Pneumonia Following Kidney Transplantation: A Case Series

**DOI:** 10.3389/fphys.2022.902465

**Published:** 2022-06-29

**Authors:** Hong-Yu Wang, Yi-Hao Li, Si-Sen Zhang, Xin Jiang, Xing-Guo Niu, Xin-Ling Qian, Cong-Yan Liu

**Affiliations:** ^1^ Department of Emergency Medicine, The Fifth Clinical Medical College of Henan University of Chinese Medicine, Zhengzhou, China; ^2^ Department of Emergency Intensive Care Unit, People’s Hospital of Henan University of Chinese Medicine/Zhengzhou People’s Hospital, Zhengzhou, China; ^3^ Organ Transplant Department, People’s Hospital of Henan University of Chinese Medicine/Zhengzhou People’s Hospital, Zhengzhou, China

**Keywords:** extracorporeal membrane oxygenation, kidney transplantation, *P. jirovecii* pneumonia, acute respiratory distress syndrome, application effect

## Abstract

**Objective:** To investigate the application effect of extracorporeal membrane oxygenation (ECMO) in patients with severe acute respiratory distress syndrome (ARDS) caused by *Pneumocystis jirovecii* pneumonia (PJP) after kidney transplantation.

**Methods:** This is a case series on 10 kidney transplant recipients with severe ARDS caused by PJP at the People’s Hospital of Zhengzhou, who were enrolled as the case group. A total of 17 cases of PJP diagnosed with severe ARDS without ECMO were selected as the control group. The timing and mode of ECMO support and treatment complications were summarized. The primary aim of this study was mortality and secondary was imaging and complications.

**Results:** The enrolled patients’ oxygenation index before the start of ECMO ranged from 25 to 92, and the time from admission to the start of ECMO was 1–17 days, with an average of 5.56 days. In the case group, one patient died of hemorrhagic shock due to abdominal hemorrhage, but the other nine patients were successfully weaned from ECMO. Of these patients, one died due to sepsis following weaning. The survival rate in the case group was 80.0% (8/10), and the survival rate in the control group was 35.29% (6/17). The vein–vein ECMO support time in the nine successfully weaned patients in the case group ranged from 131 to 288 h, with an average of 215.5 h. Of the eight patients who survived, deterioration of renal function after transplantation occurred in two patients, but no fatal complications occurred.

**Conclusion:** Overall, Patients with severe ARDS caused by postoperative PJP infection following kidney transplantation have a poor prognosis. The mortality was lower in patients who were treated with ECMO compared to standard care.

## 1 Introduction

Due to long-term use of anti-rejection drugs and hormones, patients who have undergone kidney transplantation are prone to opportunistic infections, especially *Pneumocystis jirovecii*. Such infections can progress to severe acute respiratory distress syndrome (ARDS) (Brakemeier et al., 2018), and extracorporeal membrane oxygenation (ECMO) therapy is often required. However, cases with severe ARDS caused by *P. jirovecii* pneumonia (PJP) following kidney transplantation are rare. Severe hypoxemia often leads to death in such patients. Application of ECMO at this time can correct hypoxemia and allow sufficient time for infection control, saving patients’ lives (Chiel et al., 2022). There are few reports on ECMO therapy for such patients, and treatment experience is limited. Therefore, this paper reviews the clinical history of patients with severe ARDS caused by PJP who received ECMO support in the organ transplantation center of the People’s Hospital of Zhengzhou and discusses ECMO’s intervention time, implementation method, and process management.

## 2 Materials and Methods

### 2.1 General Data

A total of 10 patients with severe ARDS caused by PJP following kidney transplantation who were admitted to the Department of Critical Care Medicine at the People’s Hospital of Zhengzhou from April 2018–May 2021 were enrolled in the case group. There were eight males and two females aged 31–67 years, with a mean age of 47.5 years. Extracorporeal membrane oxygenation technology had not been implemented in the hospital before March 2018. A total of 17 cases of PJP diagnosed with severe ARDS from March 2014 to March 2018 were selected as the control group (Table 1), comprising fourteen males aged 28–68 years, with a mean age of 45.5 years. None of the patients in the control group were treated with ECMO. There was no significant difference in the baseline characteristics or pO_2_/FiO_2_ between the case group and the control group. Following transplantation, the patients were given the anti-rejection drugs mycophenolate mofetil (0.5 g twice daily) and prednisone (10 mg once daily). They were also treated with tacrolimus, the plasma concentration of which was maintained at 6–8 ng/ml. All patients were confirmed to have PJP. In the case group, three cases were confirmed by detecting trophozoites stained with Gomori’s methenamine silver, and seven cases were confirmed by metagenomic next-generation sequencing of alveolar lavage fluid (Zhang et al., 2021). All patients underwent pulmonary high-resolution computerized tomography (CT) examination. The machines providing ECMO support were produced by the Sorin Group, and the ECMO pipeline system was produced by Maquet. The study was conducted in accordance with the principles of the Declaration of Helsinki, and the study protocol was approved by the hospital’s ethics committee. Due to the retrospective nature of the study, the requirement of patient consent for inclusion was waived.

**TABLE 1:** The basic clinical features, disease outcome of the control group.

Case Number	Age (year)	Gender	Weight (Kg)	Height (cm)	Clinical outcome	Oxygenation index (pO_2_/FiO_2_)
1	59	Male	72	170	Died	50
2	40	Male	51.5	160	Survived	70
3	46	Male	55	161	Died	68
4	40	Male	68	168	Died	56
5	35	Male	60	167	Died	38
6	55	Male	72	175	Survived	68
7	43	Male	48	167	Died	50
8	31	Female	63	168	Died	49
9	54	Male	51	160	Survived	68
10	44	Male	58	171	Died	45
11	53	Male	59	163	Died	43
12	51	Male	82	175	Survived	76
13	50	Male	42	164	Died	56
14	28	Female	72	172	Survived	64
15	68	Female	57	167	Survived	61
16	31	Male	67	170	Died	67
17	45	Male	81	172	Died	48

The diagnosis of ARDS was based on the 2012 Berlin definition criteria (Ranieri et al., 2012), which are as follows: ① acute onset or exacerbation of respiratory symptoms within 1 week; ② respiratory failure that cannot be explained by cardiac dysfunction or fluid overload, with pulmonary ultrasound ruling out pulmonary edema with high hydrostatic pressure; ③ X-ray chest radiographs suggesting bilateral infiltration shadows that cannot be fully explained by pleural effusion, nodules, or masses; and ④ oxygenation indices of 200–300, 100–200, and <100, indicating mild, moderate, and severe cases, respectively. In this study, all patients met the diagnostic criteria for severe ARDS, and their oxygenation index was less than 100.

### 2.2 Treatment Procedure

#### 2.2.1 General Treatment After Onset

The disease progression was similar in the 10 patients in the case group. They were admitted to the hospital with complaints of fever, dry cough, and progressive dyspnea. Following a poor response to conventional oxygen therapy, they were transferred to an intensive care unit. On physical examination, the respiratory sounds in both lungs were coarse, and no obvious wet or dry rales or signs of respiratory distress were heard. Chest CT showed diffuse, patchy ground-glass shadows in both lungs. Alveolar lavage fluid was taken for examination, and metagenomic next-generation sequencing and Gomori’s methenamine silver staining were used to confirm the diagnosis. According to the patients’ medical history, clinical manifestations, and imaging characteristics, PJP infection was suspected. An intravenous drip of sulbactam and cefoperazone (3.0 g q8h) and compound sulfamethoxazole tablets (0.96 g q6h) were given. A twice-daily intravenous transfusion of ganciclovir (10 mg/kg/d) was also used to prevent cytomegalovirus infection, and methylprednisolone was given to prevent inflammation. Immunosuppressive drugs were suspended, and the patients underwent nutritional support, analgesia, and sedation. If the patients were still experiencing respiratory distress under non-invasive ventilation conditions, their treatment was changed to include invasive ventilation methods, and further analgesia and sedation were given. Prone ventilation was performed when necessary, and muscle relaxants were administered if respiratory distress was difficult to control to avoid lung injury due to excessive transpulmonary pressure.

#### 2.2.2 Establishment Process of Extracorporeal Membrane Oxygenation

The ECMO perfusion occurred through the right internal jugular vein, and the drainage occurred from the femoral vein on the opposite side of the transplanted kidney. Percutaneous catheterization was guided by conventional ultrasound, and saline was used to pre-flush the ECMO pipeline system. A 21F cannula model was used at the drainage end, and a 17F model was used at the perfusion end. Before catheterization, a 50- to 100-U/kg intravenous injection of common heparin was given to prevent intravascular coagulation. After successful catheterization, the position of the catheter tip was determined by ultrasound to minimize recirculation, and the maximum flow tested was 4.8–5.7 L/min. The ECMO gas flow was adjusted according to the arterial blood gas analysis results, and a general ventilation flow ratio of approximately 1:1 was maintained.

During the ECMO operation, the platelet count was maintained above 50 × 10^9^/L, the hematocrit above 40%, and the fibrinogen above 1.5 g/L. The initial ECMO flow rate was set to high. When the circulation and oxygenation were stabilized, the arterial blood gas, hemodynamics, and pulmonary conditions were gradually reduced to optimal levels. During ECMO diversion, anticoagulation was performed *via* continuously pumping common heparin to maintain the whole-blood activated clotting time (ACT) at 180–200 s. The activated prothrombin time was maintained at 50–70 s. Two days before ECMO transfer, routine blood tests, ACT, and clotting tests were performed every 6 h. After the goal of stable coagulation was achieved, the blood routine parameters and coagulation function were reviewed every 8 or 12 h. The risk of thrombosis was assessed by daily evaluation of the D-dimer levels and platelet counts.

#### 2.2.3 Other Treatment Measures During Extracorporeal Membrane Oxygenation Bypass

A protective lung ventilation strategy was adopted during ECMO bypass. The ventilator was set to pressure control mode, and its oxygen concentration was 30%–40%. The positive end-expiratory pressure was 8–10 cm H_2_O, the pressure was controlled at 10–15 cm H_2_O, and the average airway pressure was kept below 30 cm H_2_O. In the case group, one patient died of hemorrhagic shock due to abdominal hemorrhage, other nine patients were treated with prone ventilation for more than 12 h a day during ECMO bypass. Restrictive fluid resuscitation and continuous renal replacement therapy (CRRT) were performed if necessary. A total of seven patients received CRRT treatment. In all CRRT connections, an ECMO membrane was used to join the CRRT pipeline. To reduce pulmonary edema, the patients were sufficiently dehydrated according to the blood pressure. For analgesia and sedation, the patients were continuously pumped with sufentanil combined with midazolam; a muscle relaxant was administered if respiratory distress was evident, and a low dose of sufentanil combined with dexmedetomidine was pumped at a later stage. Chest X-rays were checked daily for changes in lung permeability.

#### 2.2.4 Evacuation Standard of Extracorporeal Membrane Oxygenation

After the application of vein–vein (VV)-ECMO, the patients’ respiratory function generally began to recover within 3–4 days and gradually improved thereafter. The chest radiographs showed increased brightness, and the oxygenation index increased. The evacuation time was comprehensively analyzed according to each patient’s overall condition, chest X-ray, chest CT, arterial blood gas values, and lung compliance. If the ventilator’s oxygen concentration was 40%, the pressure support was 15 cm H_2_O, and the positive end-expiratory pressure level was 5–8 cm H_2_O, the ECMO air source was withdrawn. After half an hour of “idling,” an arterial blood gas analysis was performed. When the partial oxygen pressure was greater than 80 mm Hg and the partial carbon dioxide pressure lower than 45 mm Hg, the ECMO withdrawal was considered successful.

## 3 Statistical Analysis

The SPSS 13.0 statistical software was used for the analysis. The mortality of the case and control groups was compared using Fisher’s exact test, and *p* < 0.05 was considered statistically significant.

## 4 Results

### 4.1 Basic Information, Disease Outcomes, and Extracorporeal Membrane Oxygenation Operation

General information regarding the 10 patients in the case group is shown in Table 2. The average weight was 65.4 kg, the average age was 47.5 years, and the average height was 167.7 cm. In the control group, the average weight, age, and height of the 17 patients were 69.2 kg, 49.4 years, and 163.6 cm, respectively. The time from surgery to VV-ECMO installation was 4–157 months, with an average of 44.7 months. The oxygenation index before ECMO was 25–92. The time from admission to the placement of ECMO assistance ranged from 1 to 17 days, with an average of 6 days. The duration of mechanical ventilation prior to ECMO placement ranged from 1 to 14 days, with an average of 3.2 days. Before receiving ECMO, three patients in the case group developed mediastinal emphysema or subcutaneous emphysema.

**TABLE 2:** The basic clinical features, disease outcome and ECMO operation of the patients.

Case Number	Age (year)	Gender	Weight (Kg)	Height (cm)	Successful weaning or not	Clinical outcome	Oxygenation index before ECMO treatment (pO_2_/FiO_2_)	Serum creatinine before ECMO	Serum creatinine on discharge	Time of onset after kidney transplantation (months)	Time from admission to initiation of ECMO (days)	Mechanical ventilation time before ECMO (days)	ECMO running time (h)
1	37	Male	73	170	Yes	Survived	40	73	170	4	1	2	270
2	41	Male	48	164	Yes	Died	32	48	—	136	8	1	131
3	54	Female	53	160	Yes	Survived	65	53	160	4	1	2	230
4	54	Female	62	155	Yes	Survived	43	62	155	26	3	2	231
5	55	Male	61	175	Yes	Survived	44	61	175	4	5	1	288
6	31	Male	71	170	No	Died	65	71	—	86	6	2	182
7	66	Male	80	170	Yes	Survived	58	80	170	25	4	4	165
8	37	Male	67	163	Yes	Survived	51	67	163	2	5	3	261
9	33	Male	68	178	Yes	Survived	73	68	178	3	17	14	148
10	67	Male	71	172	Yes	Survived	42	208	Dialysis	157	10	1	322

Eight of the patients in the case group were evacuated from ECMO before continuing mechanical ventilation for 3–6 days. The other two patients were evacuated from mechanical ventilation first. After 3 or 4 days of “sober” ECMO assistance, the ECMO application was withdrawn. One patient died of hemorrhagic shock due to abdominal hemorrhage, but the other nine patients were successfully evacuated. One patient subsequently died due to sepsis following weaning, so in total, eight patients survived. Regarding the nine patients who were successfully weaned, the VV-ECMO support time ranged from 131 to 322 h, with an average of 204.6 h.

### 4.2 Comparison of Mortality Before and After Extracorporeal Membrane Oxygenation Treatment

The overall survival rate of the patients in the case group was 80.0%. Of the 17 patients in the control group, six survived, and the survival rate was 35.29%. The mortality rate in the control group was as high as 64.71%. Since ECMO therapy had yet to be implemented in the hospital when they were admitted, none of the patients in the control group received ECMO. There was a statistical difference in the survival rate between the two groups (*χ* ^*2*^ = 3.409, *p* = 0.046), suggesting that the chances of patient survival following ECMO treatment are significantly improved.

### 4.3 Imaging Findings and Etiological Findings of Patients

**FIGURE 1:**
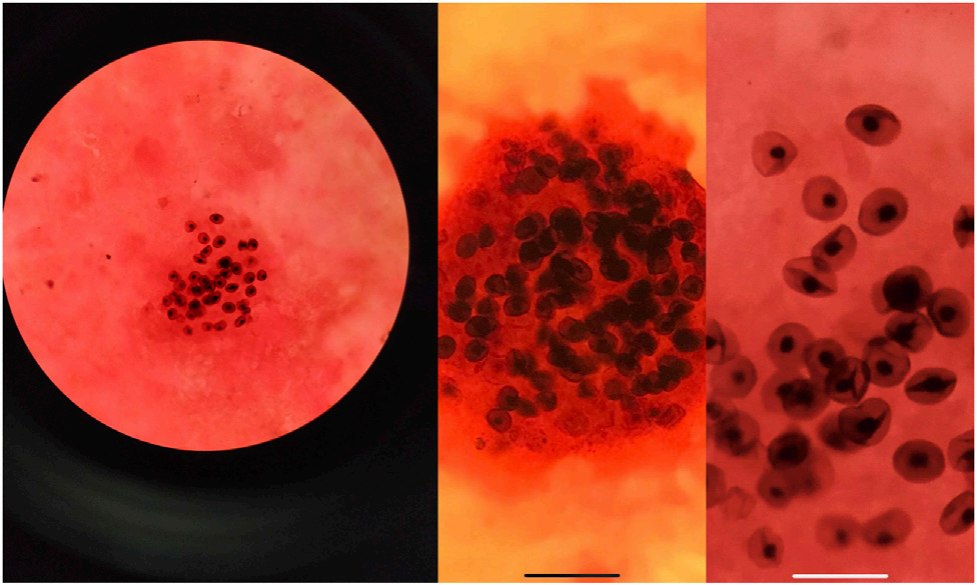
The alveolar lavage fluid was collected for examination, and the images of *P. jirovecii* pneumonia trophozoites were obtained by Gomori’s methenamine silver staining.

**FIGURE 2:**
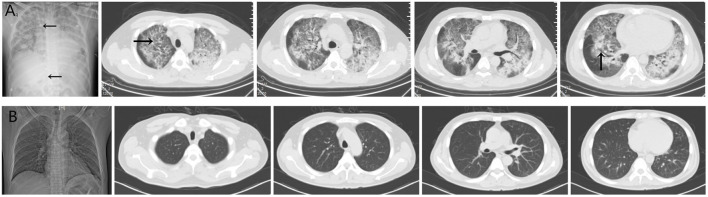
**(A)** The imaging findings of case 1 showed diffuse and uniform exudation in both lungs (→) with less pleural involvement and local consolidation with the air bronchogram sign (↑). **(B)** After extracorporeal membrane oxygenation treatment, the lung imaging showed significant improvement, increased permeability, and improvement of the infiltration shadow.

**FIGURE 3:**
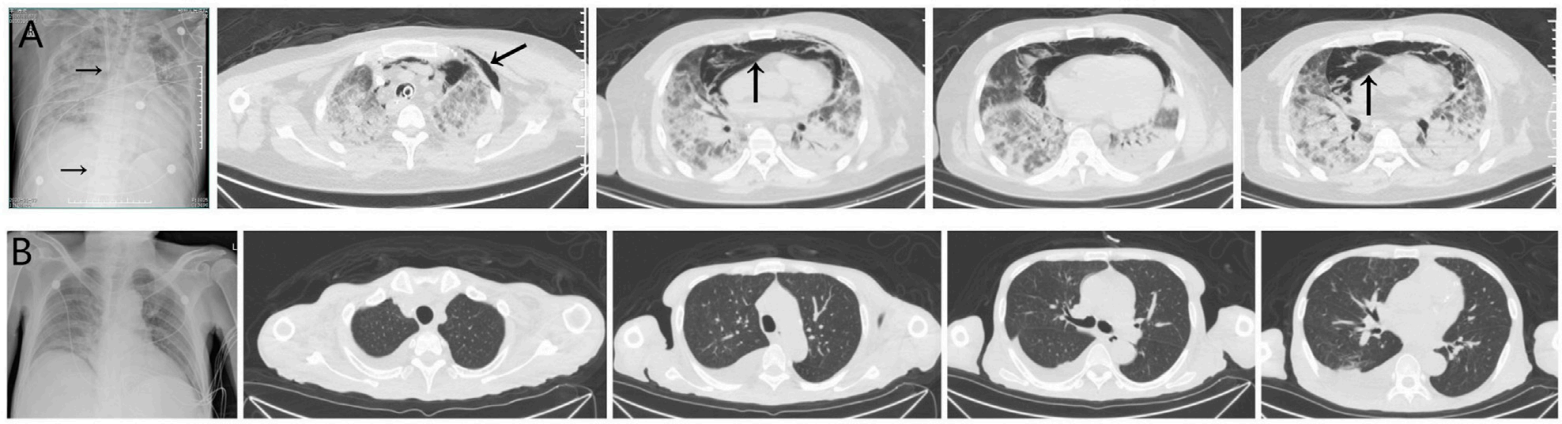
**(A)** The imaging findings of case 5 showed diffuse exudation in both lungs, especially in the lower lungs, local consolidation with the air bronchogram sign, and obvious subcutaneous and mediastinal emphysema (↑). The vein–vein extracorporeal membrane oxygenation pipe was in a good position (→). **(B)** After extracorporeal membrane oxygenation treatment, the lung imaging showed significant improvement and increased permeability, and the mediastinal emphysema disappeared.

**FIGURE 4:**
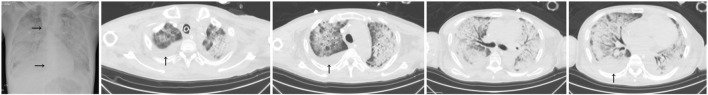
The imaging findings of case 6 (deceased) showed diffuse exudation and extensive consolidation in both lungs, with more obvious lesions in the lower lung than in the upper lung, the air bronchogram sign, and right pleural effusion (↑). The vein–vein extracorporeal membrane oxygenation pipe was in a good position (→).

Three cases of PJP infection in the case group were confirmed by Gomori’s methenamine silver staining and microscopic observation of trophozoite growth, and seven cases were confirmed by metagenomic next-generation sequencing (Figure 1). Bedside chest radiography was performed routinely for all patients, and all patients were transported to the imaging department for pulmonary high-resolution CT examination during ECMO treatment. The imaging findings included diffuse, uniform exudation in both lungs, with less pleural involvement and the air bronchogram sign, and obvious subcutaneous mediastinal emphysema. Diffuse exudation was more obvious in the lower lungs than in the upper lungs. After VV-ECMO was removed successfully, lung imaging showed significant improvement, including increased permeability and improvement of the infiltration shadow (Figures 2, 3, 4).

### 4.4 Extracorporeal Membrane Oxygenation-Related Complications

Patient complications during ECMO support included three cases of puncture point bleeding, two cases of gastrointestinal bleeding, one case of respiratory tract bleeding, one case of pleural bleeding, and one case of retroperitoneal hematoma formation. In addition, there was one case of ECMO-catheter-related bloodstream infection and one case of lower-extremity venous thrombosis. No ECMO system complications, such as equipment failure or air embolus, occurred.

## 5 Discussion

*P. jirovecii* pneumonia is a fatal opportunistic infection caused by *P. jirovecii* and is common in patients undergoing tumor chemotherapy and solid organ transplantation and those with autoimmune diseases and various types of congenital or acquired immune deficiency (White et al., 2017). In patients without Human Immunodeficiency Virus (HIV), PJP has a more acute onset, a severer inflammatory response, and more obvious hypoxemia than in those with HIV (Ricciardi et al., 2017). The mortality rate can reach 50% if ARDS progresses and even 100% if severe ARDS, pneumothorax, or other complications occur (Festic et al., 2005). *P. jirovecii* pneumonia infections are not uncommon following organ transplantation due to immunosuppressant treatment. Generally, the incidence of PJP 3–6 months after kidney transplantation is high, and the success rate of treatment for these patients is low once severe ARDS develops (Lee et al., 2017). If the lung CT of postoperative kidney transplant patients shows diffuse ground-glass shadows in both lungs, the possibility of PJP infection should be considered. Timely collection of pulmonary alveolar lavage fluid should be performed to carry out next-generation sequencing or Gomori’s methenamine silver staining to confirm PJP (Azoulay et al., 2009).

As an external life support system technology, ECMO is rarely used in the treatment of PJP infection following solid organ transplantation despite severe pneumonia being common in such cases (Nureki et al., 2020). Although VV-ECMO has been reported to treat severe ARDS post-transplantation, the number of relevant studies is minimal, and all are case reports (Wu et al., 2013). The People’s Hospital of Zhengzhou completes nearly 400 kidney transplantation surgeries every year, and there have only been 10 cases of patients with PJP infection complicated with severe ARDS and treated by VV-ECMO. In the past, the hospital has demonstrated poor efficacy in treating patients with severe ARDS caused by PJP with mechanical ventilation alone. From March 2016 to March 2018, ECMO was not utilized, and the mortality rate of patients receiving mechanical ventilation alone reached 64.71%. Since April 2018, the hospital has applied active ECMO support combined with mechanical ventilation, and the mortality rate has decreased significantly. In this study, the weaning success rate for the 10 patients in the case group was 90.0%, and the mortality rate was 20.0%, suggesting that ECMO has great advantages in the treatment of severe ARDS caused by PJP. A positive therapeutic effect was achieved, and experience in using ECMO was gained.

In terms of the timing of ECMO intervention, most centers currently follow the recommendations of the Extracorporeal Life Support Organization. A lung-protective ventilation strategy is adopted when the oxygenation index is less than 100, the respiratory rate is greater than 35 times/min, and the blood pH is less than 7.2 (Ślusarz et al., 2019). Based on the current study, it is suggested that VV-ECMO intervention should be initiated as early as possible when the oxygenation index is less than 150 without strictly following the six-step ventilation strategy for severe ARDS (Fan et al., 2018). If the patient’s pulmonary symptoms deteriorate rapidly within 1–2 days or they experience mediastinal or subcutaneous emphysema, then ECMO assistance can be initiated when the oxygenation index is less than 200; this has not been reported in previous literature (Schmidt et al., 2018; Rilinger et al., 2019). Active intervention is important, as PJP is mainly caused by extensive exudation in both lungs and progresses rapidly. Respiratory distress is prone to causing pneumothorax, mediastinal emphysema, alveolar injury, and other complications, leading to greater treatment difficulties (Arichi et al., 2009). For example, a patient examined in this study (case 5) did not receive ECMO support initially and was treated with non-invasive ventilation for 10 days. Pulmonary bacterial infection, subcutaneous and mediastinal emphysema, and other complications occurred, and the patient required ECMO assistance for recovery. When ECMO is introduced at an early stage, there are fewer instances of ventilator-related lung injury, which is also conducive to faster lung recovery after ECMO treatment and earlier withdrawal of ECMO support. Therefore, early and aggressive ECMO support is recommended to reduce ECMO-related complications and medical costs and improve the treatment success rate.

Compared with cases of ARDS caused by other diseases, a strict restrictive fluid resuscitation strategy is recommended for PJP cases following ECMO assistance, and ECMO combined with CRRT dehydration should be utilized when necessary. Since the main pathological changes of PJP are alveolar exudation and the collapse of the alveoli at the base of the lung, the purpose of reducing the fluid load is to reduce pulmonary edema and improve oxygen levels (Fan et al., 2018). Reducing mechanical ventilation support parameters to achieve lung rest following ECMO support can also lead to alveolar edema. Restricted fluid resuscitation is required to reduce pulmonary edema (Liang and Chen, 2022). However, since PJP cases mainly present with alveolar exudation, the alveolar collapse at the lung base is more severe. Therefore, it is recommended that attention be paid to the need for prone ventilation after ECMO implementation; the prone ventilation time should exceed 12 h a day (Guérin et al., 2020). In this study, all nine patients who were successfully weaned from ECMO underwent prone ventilation, which can promote earlier recovery in PJP cases.

The ECMO withdrawal time in this study was typically around 7 days. At present, diseases requiring VV-ECMO–assisted treatment generally require 10–15 days of therapy, and the overall success rate of the treatment is less than 50% (Fan et al., 2016). Compared with previous research, the ECMO duration in this study was shorter, and the therapeutic effect was better (the weaning success rate was 90%), suggesting that VV-ECMO has a better therapeutic effect on PJP than on other diseases. Due to the short duration of ECMO, the weaning can take place as soon as possible once the required weaning standard is achieved. The intervention and withdrawal principle of “early boarding and early weaning” can significantly reduce ECMO-related complications such as infection, bleeding, thrombosis, and malnutrition. Therefore, for patients with PJP infection following kidney transplantation, active ECMO adjuvant therapy is recommended. Bleeding is the most common complication of ECMO due to the need for heparin anticoagulation during treatment. In this study, there were many bleeding complications. However, most were not serious and could be relieved by specific treatments, such as heparin dosage adjustment, infusion of fresh frozen plasma or platelets, and local compression.

There are two main shortcomings of this study. First, the number of cases investigated was small, and the cases stemmed from a single location. As the disease studied is rare in clinical practice, a collaborative study involving multiple transplant centers should be conducted to enlarge the case number. Second, this was a retrospective study that lacked a proper control group. Therefore, it is recommended that a satisfactory control group be used for future studies.

In conclusion, in this case series of patients with severe ARDS caused by postoperative PJP infection following kidney transplantation have a poor prognosis, the mortality was lower in patients who were treated with ECMO compared to standard care.

## Data Availability

The original contributions presented in the study are included in the article/Supplementary Material, further inquiries can be directed to the corresponding author.
